# Special Issue: Molecular Mechanisms and Treatment of Retinal Diseases

**DOI:** 10.3390/ijms27031376

**Published:** 2026-01-30

**Authors:** Deokho Lee, Livio Vitiello

**Affiliations:** 1 The Korean Institute of Nutrition, Hallym University, Chuncheon 24252, Republic of Korea; 2 Eye Unit, “Luigi Curto” Hospital, Azienda Sanitaria Locale Salerno, 84035 Polla, SA, Italy

The retina, one of the most metabolically active tissues in the human body, is susceptible to a wide range of injuries. Retinal degeneration can occur due to natural aging, acute and chronic inflammation, vascular dysfunction, or oxidative stress [1,2,3,4], and it remains a major cause of vision loss and visual impairments worldwide. Metabolic regulation has recently been considered as a strategy for suppressing retinal degeneration. Many studies have suggested that supplementation with targeted nutrients may reduce oxidative stress, modulate inflammation, and preserve retinal integrity [5,6,7]. Moreover, innovative therapies aimed at restoring or preserving vision, such as cell-based treatments and advanced drug delivery systems, have emerged in recent years [8,9,10,11,12].

The aim of this Special Issue was to collect relevant research focused on nutritional interventions, optometric management, regenerative therapies, drug delivery innovations, and digital health applications to reduce or block retinal degeneration in metabolic diseases. By showcasing cutting-edge research and clinical insights, we hope to inspire new therapeutic strategies to protect vision and enhance quality of life worldwide. This Special Issue comprises three research papers and two reviews, and a brief description of their content is provided below.

Glaucoma research: Bodganov et al. examined whether sitagliptin eye drops could prevent corticosteroid-induced experimental glaucoma in mice [13]. Through immunohistochemical analyses, sitagliptin was shown to exert promising preventive effects on retinal ganglion cells and oligodendroctyes in the damaged retina and optic nerve head, with anti-inflammatory effects also detected in the latter. Glaucoma is one of the leading causes of visual impairments worldwide, and treatment options for this pathologic condition are limited [14,15,16,17,18]. Therefore, this study provides a novel neuroprotective approach for safe and effective glaucoma treatment. Sitagliptin’s therapeutic effects have been examined in other pathologic conditions in the retina. Simó et al. suggested that sitagliptin could prevent diabetes-evoked vascular leakage in human retinal endothelial cells (HRECs) and ARPE-19 cells [19]. Ramos et al. showed that sitagliptin eye drops could prevent retinal histologic damage, abnormal vasodilation, vasodegeneration, inflammation, and oxidative stress in experimental retinopathy in rats [20]. Taken together, sitagliptin could be considered an effective treatment for various retinal diseases.

Age-related macular degeneration (AMD) research: Dörschmann et al. established fundamental assay protocols for porcine single-eye retinal pigment epithelium (RPE), which was aimed at modulating inflammation and oxidative stress [21]. The use of experimental RPE models is crucial for understanding and unraveling the pathologic mechanisms of AMD and developing new therapeutics at the preclinical stage [22,23,24,25]. They found that, for oxidative stress experiments, effective LD_50_ can be obtained following 24 h stimulation with 25 µM erastin one week after cell preparation in 5% serum cultures, in the absence of plate coating. Poly-D-Lysine coating is required when gene expression changes are checked between experimental groups. For inflammation experiments, three days of 1 µg/mL LPS stimulation are needed to effectively induce cytokine activation with 5% serum on uncoated plates. Lastly, transwell plates are not a good option for assessing cytokine secretion. Although various RPE culture protocols have been introduced [26,27,28,29,30,31], there are still several limitations related to the differentiation methods and the challenges of keeping morphological/functional/structural properties close to those found in vivo. More stable and successful cell cultures and experimental settings are warranted.

Gyenes et al. investigated the duration of good visual acuity during anti-vascular endothelial growth factor (VEGF) treatment for AMD in regular clinical conduct [32]. VEGF is a key molecule that induces neovascularization in AMD, and many anti-VEGF drugs have been introduced for therapeutic purposes [33,34,35,36,37,38,39,40]. Based on a retrospective study involving a large database, anti-VEGF compounds targeting various domains were found to be markedly advantageous for treating AMD, especially when these agents are required for a longer period; this was supported by comparative analyses between aflibercept [41,42,43] and ranibizumab [44,45,46,47]. Although the limitations listed in the paper should be addressed, this study could provide insight into the effectiveness and safety of anti-VEGF therapy in a patient-centered healthcare system.

Diabetic retinopathy research: Gettinger et al. provided an overview of current pathophysiology and experimental models for diabetic retinopathy [48]. Although many review articles have described diabetic retinopathy and its pathophysiology [49,50,51,52,53], this new article has a novel approach, combining the pathophysiology of DR and relevant preclinical experiment models in vivo and in vitro. In particular, an accelerated diabetic retinopathy model has been newly introduced in the form of streptozotocin (STZ)-induced diabetic mice [54,55] with carotid artery occlusion-induced retinal ischemia [56]. As this model can exhibit accelerated inflammation and vascular dysfunction under diabetic ischemic conditions, diabetic retinopathy research related to relevant pathophysiology and novel drug screening is able to be facilitated.

Ocular hemorrhage: Eder et al. introduced sub-internal limiting membrane (sub-ILM) hemorrhage [57,58,59,60], a distinct pre-retinal bleeding entity where blood is stacked between the ILM and the retinal nerve fiber layer, and they further reviewed available treatment and management for this hemorrhage [61], using simple and continuous observations, and including neodymium-doped yttrium aluminum garnet laser ablation, pneumatic displacement, and vitrectomy. Their findings can be used to boost the development of evidence-based and consensus-driven guidelines to optimize visual acuity and prevent further retinal damage caused by this case.

In conclusion, this Special Issue presents a collection of research and review articles that advance our understanding of molecular mechanisms and treatment of various retinal diseases. We hope that these contributions will be highly influential, informing future translational research by assisting with the development of various novel therapeutic interventions for improved disease control and management.
